# Exploring the Impact of Various Wooden Barrels on the Aromatic Profile of Aceto Balsamico Tradizionale di Modena by Means of Principal Component Analysis

**DOI:** 10.3390/molecules29112647

**Published:** 2024-06-04

**Authors:** Caterina Durante, Lorenzo Morelli, Veronica D’Eusanio, Lorenzo Tassi, Andrea Marchetti

**Affiliations:** 1 Department of Chemical and Geological Sciences, University of Modena and Reggio Emilia, 41121 Modena, Italy; caterina.durante@unimore.it (C.D.); lmorelli@unimore.it (L.M.); veronica.deusanio@unimore.it (V.D.); andrea.marchetti@unimore.it (A.M.); 2 National Interuniversity Consortium of Material Science and Technology (INSTM), 50121 Florence, Italy; 3 Interdepartmental Research Center BIOGEST-SITEIA, University of Modena and Reggio Emilia, 42121 Reggio Emilia, Italy

**Keywords:** solid-phase microextraction, gas chromatography, vinegar, volatile organic compounds, chemometrics analysis

## Abstract

The study examines the unique production process of Aceto Balsamico Tradizionale di Modena PDO (ABTM), emphasizing its complex phases and the impact of raw materials and artisanal skill on its flavor characteristics. Analytical tests focused on the volatile composition of vinegars from different wood barrels at different aging stages, using solid-phase micro-extraction (SPME) coupled with gas chromatography, either with mass spectrometry (GC/MS) or flame ionization detector (FID). Multivariate analysis, including principal component analysis (PCA), was employed to investigate the presence of peculiarities among the volatile profiles of samples of different barrel origin. The research focuses on characterizing the volatile composition of vinegars sourced from individual wood barrels, such as Cherry, Chestnut, Mulberry, Juniper, and Oak. Although it was not possible to identify molecules directly connected to the woody essence, some similarities emerged between vinegar samples from mulberry and cherry barrels and between those of juniper and oak. The former group is characterized by analytes with high molecular weights, such as furfural and esters, while the latter group shows more intense peaks for ethyl benzoate. Moreover, ethyl benzoate appears to predominantly influence samples from chestnut barrels. Due to the highly complex production process of ABTM, where each battery is influenced by several factors, this study’s findings are specific to the current experimental conditions.

## 1. Introduction

Italy is the European country with the largest number of products certified as Protected Designation of Origin (PDO), Protected Geographical Indication (PGI), Traditional Specialties Guaranteed (TSG), or classified as organic [1]. The Emilia-Romagna region plays a leading role in driving the economic impact through the sale of food products distinguished by these geographical indications. Among these, the province of Modena makes a substantial contribution with its numerous food products with protected designations. Among these, Aceto Balsamico Tradizionale di Modena PDO (ABTM) stands out as a product with exceptional quality and reputation. While ABTM may have a lower economic impact and be less affordable than its substitute Aceto Balsamico di Modena PGI (ABM), its superior quality and distinctiveness make it a product of greater value. ABM production allows for the inclusion of wine vinegar (minimum 10% *v*/*v*) and caramel color (up to 2% *v*/*v*) [2]. In contrast, ABTM is restricted to using only cooked grape must. Notably, ABTM is distinguished by its commitment to exceptional quality, as it undergoes maturation and aging in distinctive wooden barrels of various sizes for at least 12 years [3]. This meticulous process contributes to the superior quality and nuanced flavor profile that sets Aceto Balsamico Tradizionale di Modena apart.

When it comes to valorizing food products, comprehensive knowledge and in-depth analysis of the production process are essential. The ABTM supply chain is uniquely characterized, dynamic, and deeply rooted in the traditions of the territory. This is evident in the resulting vinegar, which is rich in flavors and aromas and possesses an intricate complexity defining its exceptional quality [4]. Furthermore, the demand for the development of analytical tools to ensure the quality and authenticity of foods has increased significantly in recent years [5,6]. This need is particularly true for traditional foods given their high added value, which poses a higher risk of counterfeiting and subsequent marketing of “similar” products [7,8,9,10]. These imitations often originate from different regions or countries and are typically of lower quality. The concept of quality and authenticity of traditional foods is inherently complex, encompassing the identification of adulteration, adherence to traditional protocols, and determination of geographical origin. These multifaceted aspects introduce distinct sources of variability, contributing to intricate systems, both in terms of characterization and, even more significantly, in the realm of data analysis. Moreover, by definition, typical foods exhibit characteristics that vary not only over time but also within the production area and among different producers. Consequently, quality cannot merely be described in terms of conformity to a predefined set of characteristics, as is common for industrial products. Hence, the analytical methods and data analysis tools traditionally employed in food quality and process control may require re-evaluation and modification to effectively address these dynamic and diverse tasks.

The production of ABTM begins with the selection of grapes from which juice is first extracted [11]. The juice is then subjected to appropriate cooking over a direct flame in open containers, resulting in the reduced cooked must. The must, reduced through water loss, undergoes two fermentation stages: an initial partial fermentation of glucose and fructose into ethanol, driven by yeast (*Saccharomyces cerevisiae*), and a subsequent bio-oxidation process, where ethyl alcohol is converted into acetic acid by *Acetobacter*. The resulting product is referred to as “acetified must”, which serves as an authentic raw material to produce ABTM and fills the barrel batteries employed in traditional vinegar factories. Typically, a battery consists of a sequence of containers of different volumes, called barrels or casks, ranging from a minimum of 5 to a maximum of 12. The production rules provide no specific directives regarding the quantity or dimensions of the barrels. Instead, they simply stipulate that these barrels should be constructed using “classic types of wood from the area”, with reference to the province of Modena. Tradition dictated the utilization of woods such as juniper, mulberry, oak, cherry, and chestnut. The significant role of woody essence in influencing the organoleptic characteristics of vinegars, such as sherry vinegar [12], or sweet wine, such as Pedro Ximénez Sweet Wine [12], has been widely recognized and investigated. These studies allowed significant correlations among aging time, wood toasting, and the type of wood used for aging to be found.

As for ABTM, its production process is more articulated, as it involves aging not in a single wooden barrel but in a series of wooden barrels of decreasing size through the topping-up procedure [5,13]. Through this ‘dynamic’ technique, vinegar assimilates distinctive aromas and flavors, which further enhances the final product’s unique character. The commonly adopted practice involves the use of various wood types, combining the robust notes of certain woods, such as cherry, juniper, or mulberry, with the delicate aromas of other woods, namely, chestnut, ash, or oak, thus creating a condiment rich in flavor and more complex. Recently, some producers have ventured into the scope of single-wood batteries, comprising barrels obtained from a singular wood essence. The objective is to explore novel and distinctive flavors, emphasizing the pronounced and distinct fragrance of the chosen wood. This approach further accentuates the character of their products, presenting consumers with an expanded array of choices.

Numerous studies have sought to elucidate the composition of the volatile fraction in balsamic vinegar [3,14,15,16]. The existing literature extensively covers investigations into the differences between ABM and ABTM, with the main aim of distinguishing the more esteemed product (ABTM) from its commercial counterpart (ABM). The HS-SPME technique coupled with GC-MS analysis was also used to monitor the volatile fraction of different ABTM samples throughout the aging process [5] and to find correlations with their sensorial parameters [13].

Notwithstanding the extensive research carried out on balsamic vinegar, an unexplored aspect concerns the potential influence exerted by the wood used for barrel construction on the final aroma of the product. This presents a significant challenge due to the multifaceted nature of the factors that influence aroma development, including raw material selection, duration of aging, and other relevant variables. In addition, to achieve the desired organoleptic attributes of vinegar, producers predominantly use composite batteries that include barrels made from different wood types, making it uncommon to identify traditional “single-wood batteries” in which all barrels are made from a uniform type of wood.

Despite these complexities, in this study, it was possible to investigate vinegar samples coming from five batteries of different types of wood, namely chestnut, cherry, mulberry, juniper, and oak, each consisting of six barrels made from the same variety of wood. Although few samples were available, this experimental design could support the study of the precise impact of each type of wood on the final aromatic profile of balsamic vinegar, thus providing new insights into the role of wood selection for the distinct characteristics exhibited by the product. However, the findings of this study are specific to these particular samples, and therefore, are not generalizable to all ABTM production. This specificity is due to the highly complex production process of ABTM, where each battery is unique and influenced by many factors such as the raw materials and the artisanal practices. For the aim of this study, the aromatic profile of the investigated vinegar samples was analyzed by means of Headspace-Solid-Phase Micro-Extraction-Gas Chromatography-Mass Spectrometry (HS-SPME-GC/MSq). To gain an overview of the existing relationships among the aromatic profile of vinegars, aging, and the different types of wooden barrels, the obtained results were analyzed with Principal Component Analysis by considering the whole chromatographic profile as a fingerprint of each investigated sample.

This methodological strategy overcomes traditional analytical approaches, as it has the ability to consider a multitude of parameters simultaneously, rather than focusing, as is commonly done, on macro-descriptor parameters such as pH, density, viscosity, etc. The application of multivariate analysis is crucial in the study of ABTM, as it represents great support in probing and interpreting the compositional complexity of the product. In addition, through the synergistic application of multivariate analysis techniques and chromatographic aroma characterization of samples, it was possible to gain insight into the transformations that occur within the product along the aging phase of vinegar samples. Characterization of vinegars at various stages of aging, coming from different producer’s batteries, holds significant potential for gaining a deeper understanding of the phenomena occurring during the aging period. Furthermore, it can be possible to test whether in some way the type of wood used for the barrels can create unique characteristics in the aroma of the products.

## 2. Results and Discussion

### 2.1. Identification of the Volatile Compounds by HS-SPME-GC/MSq

The identified compounds for a young (barrel 3) and an aged (barrel 6) sample belonging to the juniper battery are reported in Table S1. This preliminary analysis is crucial for identifying chromatographic peaks and obtaining preliminary information on the qualitative composition of the analyzed samples.

The volatile compounds identified in the aroma of ABTM samples can be classified into four categories [17,18]:

- Molecules present in grape must;
- Molecules formed during the cooking process;
- Molecules formed during the fermentation processes, both alcoholic and acetic;
- Molecules formed inside the battery during the product aging phase.

Determining the origin of different molecular species is an extremely complex task. The metabolic processes underlying the growth of grape berries, their maturation, and the phases following the pressing of the bunches involve a series of continuous processes that intricately shape the final product [19]. The must’s cooking, alcoholic fermentation, and acetic bio-oxidation processes constitute the cycle of “outside” transformations. The “inside” transformations involve condensation processes, resulting in the formation of low molecular weight species, the hydrolysis of wooden structures constituting the internal part of the barrels, and evaporation processes with increasing concentrations of solutes. Several chemical and physical processes affect the barrels over time, including the extraction of tannic species contributing aromas and flavors to ABTM, as well as the fragmentation of wood fibers, leading to wear and tear of the barrels [20]. The battery functions as a real chemical reactor, actively contributing to the distinctive characteristics of the final product when properly nourished and maintained.

Below, we provide a description of the most significant compounds identified through HS-SPME-GC/MSq analysis.

After water, ethanol is the primary compound found in wine and is acquired through the controlled fermentation of sugars [19]. Within the ABTM supply chain, ethanol plays a fundamental role in acetic acid production through a bio-oxidation process facilitated by *Acetobacter*. After acetic fermentation, only a small quantity of ethanol persists within the product. In fact, the alcohol is partially consumed by the reaction with acetic acid to form ethyl acetate via an esterification reaction, and the rest is lost by evaporation. These phenomena accounted for the absence of ethanol in the volatile fraction of sample 6. Isoamyl alcohol and 2-methyl-1-butanol are isomers produced through alcoholic fermentation and are considered “secondary metabolites”. Sugar fermentation by yeasts, whether native or selected, generates a range of “secondary metabolites” within the liquid mass, contributing distinctive and characteristic aromatic notes to the final product. Typically, these metabolites are present in the aromatic fraction of ABTM. It has been demonstrated that the production of these compounds is linked to the type of yeast used [21]. These alcohols are subjected to evaporation and/or degradation during the aging phase, accounting for their absence in the volatile fraction of sample 6.

The presence of ethyl acetate is associated with fruity and floral notes [18]. Its concentration in the aroma is also influenced by its high volatility, which is higher than that of acetic acid. It was present in all the analyzed samples, as it is produced throughout the aging process through the esterification of acetic acid with ethanol. Ethyl butanoate, which also has a fruity aroma, is produced during alcoholic fermentation by *Saccharomyces cerevisiae*. It tends to evaporate or degrade during the aging phase, which accounts for its absence from the volatile fraction of sample 6.

Furan compounds play a crucial role in shaping the aromatic characteristics of ABTM [18,22]. Their origin is intricately tied to the grape juice cooking process and primarily involves glucose and fructose degradation. Elevated temperatures and prolonged heating times synergistically contribute to the increased furan compound content. As they are primarily produced during the cooking phase, these compounds were identified in all analyzed samples. Among them, furfural is noteworthy because of its toxicity when ingested or inhaled, necessitating monitoring of its concentration [23,24]. Its formation occurs not only during reactions involving sugars in the cooking of the must but also throughout the aging process. Several studies have highlighted the significance of controlling the cooking process to limit furfural formation in the final product. Specifically, high temperatures (exceeding 90 °C) and low water content (below 40%) contribute to an exponential furfural formation. Importantly, furfural is continuously generated during the aging phase of the product, indicating that sugar degradation persists even at ambient temperature. Unlike other furan compounds, 5-methyl furfural is non-toxic and originates from hexose sugars, predominantly glucose. It is formed through the dehydration and reduction of 5-hydroxymethyl-2-furfural.

It is crucial to acknowledge that the sensory contribution of wood to the final product is likely challenging to trace in the gas-phase composition, as it is probably imparted by high molecular weight molecules with low volatility and low vapor pressure, which are unsuitable for sampling and detection with the employed technique.

### 2.2. Evolution of the Volatile Fraction of ABTM: HS-SPME-GC/FID and Multivariate Analysis

In the following discussion, label “1” designates samples corresponding to the youngest product and label “6” to the oldest one. The label “ABTM” indicates the respective Aceto Balsamico Tradizionale di Modena PDO, available for commercialization. The labels assigned to the different producers are presented in Table 1 and explained in Section 3.1 below.

**Figure 1:**
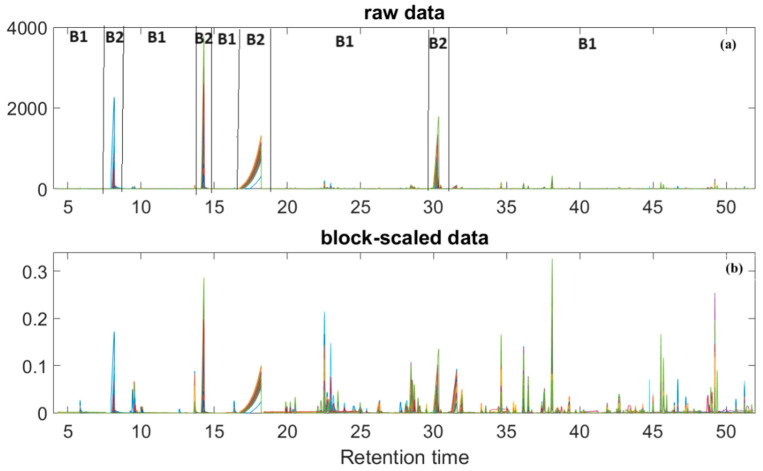
Collected GC/FID chromatograms of vinegar samples (**a**) before and (**b**) after the different data pretreatments. (**a**) Dotted lines mark the limits of the different intervals used for the scaling of the signals. Each color corresponds to a different sample. B1 and B2 are the two defined blocks.

The present study aimed to chemically characterize the aroma of vinegar samples coming from batteries consisting of single-wood barrels and to examine any potential influences of the wood used on the final aroma of ABTM. To achieve this goal, the entire chromatographic signal of each sample was considered a “signature” capable of characterizing its properties. All chromatograms were pretreated as explained in Section 3.4 and analyzed by means of Principal Component Analysis. Briefly, the chromatographic region was divided into two blocks (B1 and B2, Figure 1a) and the data were block-scaled and mean-centered. As explained, the data underwent block-scaling, to ensure uniformity among selected blocks, and a column mean-centering. The chromatograms, before and after the pretreatment procedure, with corresponding division into blocks, are reported in Figure 1.

**Figure 2:**
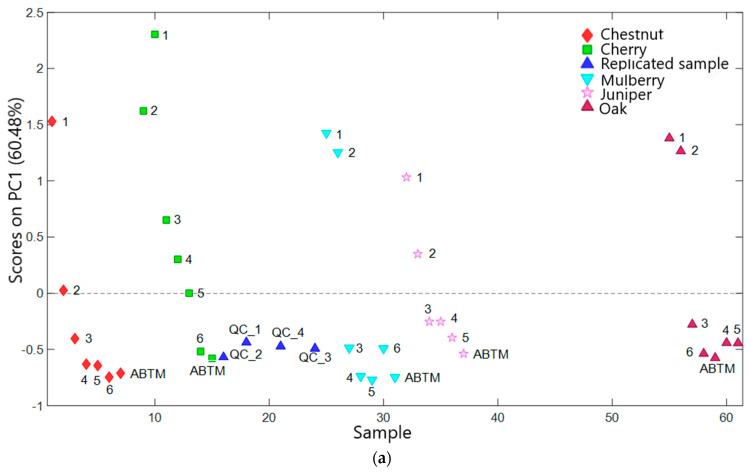

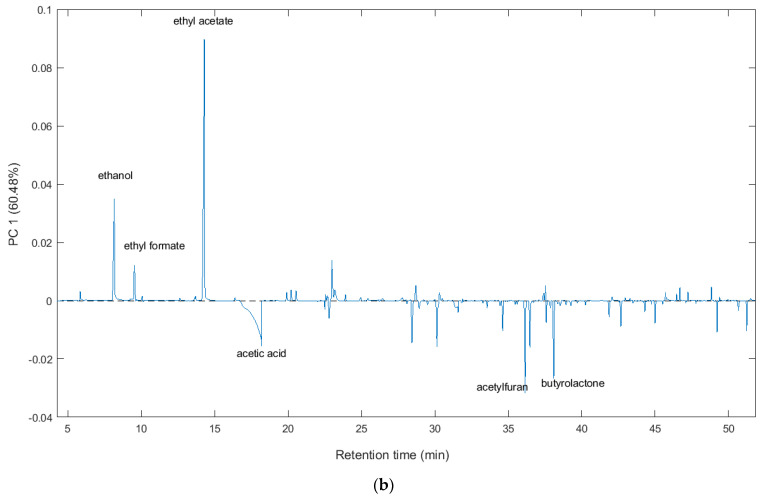
(**a**) PC1 scores plot of vinegar samples. Scores are colored considering the battery of origin. (**b**) PC2 loadings plot.

Five principal components (PCs) were utilized to develop the PCA model, explaining around 90% of the total variance (R^2^). Figure 2 displays the score and loading plots of the first principal component, PC1. Samples are represented by distinct symbols and colors corresponding to their batteries of origin. To facilitate reading and interpretation of the results, the replicate sample is labeled as “QC” followed by a number. As for the other vinegar samples, the labels refer to the barrel to which they belong (Table 1). For clarity, label 1 designates the samples from barrel 1 (the youngest samples, in terms of time in the barrel, within their battery) and label 6 the samples corresponding to barrel 6 (the oldest sample in terms of time in the barrel, within their battery). The ABTM label designates the marketable product aged at least 12 years.

**Figure 3:**
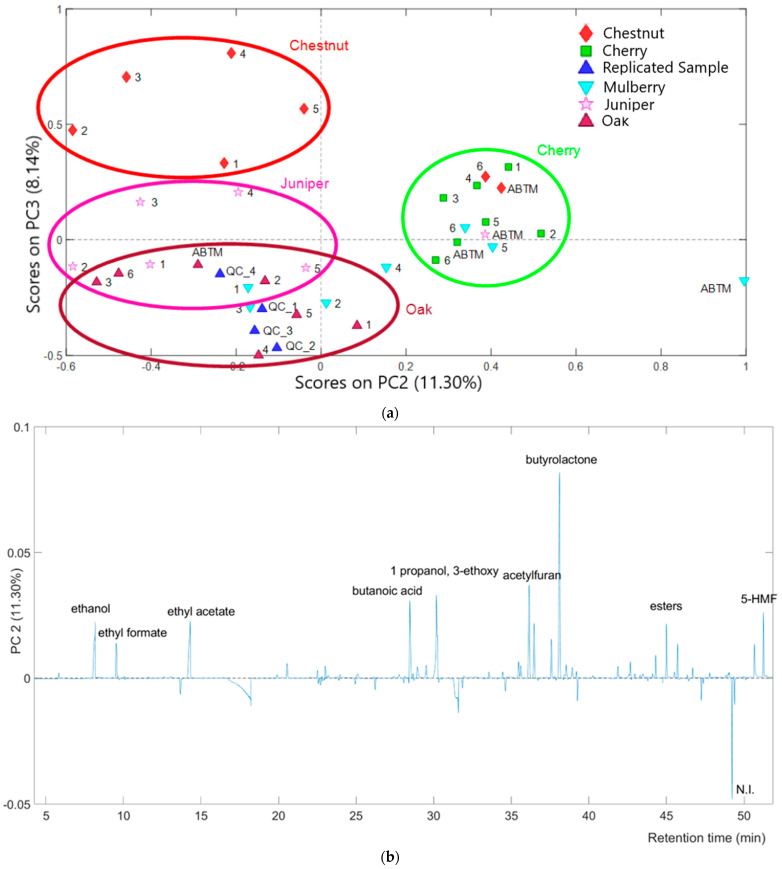

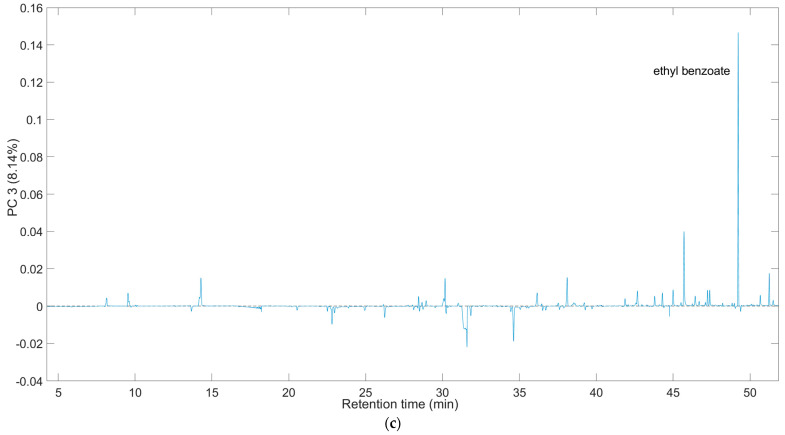
(**a**) PC2 vs. PC3 score plots of the vinegar samples. Scores are colored according to the battery of origin. Ellipses were used exclusively to improve the visualization of results. (**b**) PC2 and (**c**) PC3 loading plots. N.I.: Not identified, 5-HMF: 5-Hydroxymethylfurfural.

From a closer inspection of Figure 2a, it is possible to point out good reproducibility within the different replicates along PC1, which accounts for 60% of the variability. Furthermore, a noticeable trend was observed, indicating a decrease in the PC1 scores according to the evolution of the aging process within each battery. In particular, all sample 1s exhibit positive PC1 values, while older ones show negative values. This aligns with previous studies demonstrating a correlation between aging and changes in the aromatic fraction. Specifically, all ABTM samples from different batteries showed negative PC1 values, displaying an aromatic composition very similar to that of barrel 6. This aligns with the production technique of traditional DOP vinegar, where only the sample from the oldest barrel can be commercialized, provided it meets the production specifications. The youngest samples, from barrel number 1, exhibit similar positive PC1 values, regardless of the type of wood in the barrel. This also aligns with the product type. Indeed, since samples from barrel 1 are primarily composed of cooked must, the aroma of these samples is more influenced by the grape variety used to produce the must rather than by the type of wood in the barrel. Samples from barrel 3 onward, in all batteries, except the cherry battery, show negative PC1 values. The cherry battery stands out not only because the samples from barrels 3 and 4 show positive PC1 values but also because it shows a greater difference between the scores of samples 1 and 6. From this consideration, it could be possible to state that there is more variability in the aroma fraction between the young and aged samples from the cherry battery. Finally, it is highlighted that while for all batteries the aromas of the samples from barrels 1 and 2 are very similar, there is a difference in the chestnut battery, suggesting that chestnut may affect aroma differently than other woods in the early stages of aging. Through the PC1 loadings shown in Figure 3, it is possible to point out which compounds have a greater influence on the differences highlighted in the respective score plot.

It is noteworthy that samples with a lower degree of aging, characterized by positive PC1 values, exhibited higher concentrations of ethanol (Rt: 7.957 min), ethyl acetate (Rt: 14.384 min), and ethyl formate (Rt: 9.548 min), as indicated by positive loadings of PC1. Significantly, as the product aged, the concentration of ethanol notably decreased. Samples showing a higher degree of aging, indicated by negative PC1 values, were mainly distinguished by an increased presence of furan compounds and other compounds with higher molecular weights. These results confirm earlier research [5], providing additional evidence for the significance of chemical constituents in distinguishing vinegar samples based on their aging process. These constituents include varying levels of acetic acid, ethyl acetate, and ethanol—key products of alcoholic fermentation and bio-oxidation—as well as furfurals and other minor compounds.

Figure 3a reports the score plot of PC2 vs. PC3. While the first principal component is primarily influenced by the aging evolution of vinegar samples within their respective barrels, further insights into the differences among samples based on the type of wood barrels could be obtained through the analysis of principal component scores plots of PC2 and PC3. By focusing on samples that have spent more time in the barrels (barrels 4, 5, and 6), similarities emerge between mulberry and cherry samples, characterized by positive PC2 values, and between juniper and oak, with negative PC2 and positive PC3 values. Samples from the chestnut battery stand out from the others with negative PC2 values and positive PC3 values, while the most aged chestnut sample shows similarities with the mulberry and cherry samples.

The combined analysis of the PC2 and PC3 scores plot with the respective loadings plot (Figure 3b,c) allowed samples to be differentiated based on the presence and abundance of specific volatile compounds. Samples from batteries with cherry barrels appear enriched in high molecular weight compounds, such as esters (Rt from 40 to 46 min), butyrolactone, and furan compounds (Rt: 35.277 min, 38.802 min, and 30.559 min), most with PC2 positive loadings values (Figure 3b). Oak samples present an opposite trend compared to the aforementioned samples. They likely have a lower abundance of high molecular weight compounds and potentially lower content of ethyl benzoate (positive PC3 loading value, Figure 3c) On the other side, chestnut samples might have a higher content of the latter compound; this finding is supported by the results of earlier work [25] in which the same compound, which confers a distinct fruity and floral odor, was found as a characteristic analyte in a traditional fermented glutinous rice drink supplemented with chestnuts.

However, before asserting the presence of the highlighted analyte as a key compound in the aging of the vinegar in a chestnut battery, it will be necessary, for example, to extend the research to the analysis of the volatile fraction of the chestnut wood used for the barrels. Indeed, while there are detailed investigations on the aroma of traditional balsamic vinegar samples aged in barrels made of different woods, there is a lack of information on the volatile fraction of the barrels before being filled with cooked must or aged vinegar samples. An assessment in this direction will certainly be carried out in future studies.

**Figure 4:**
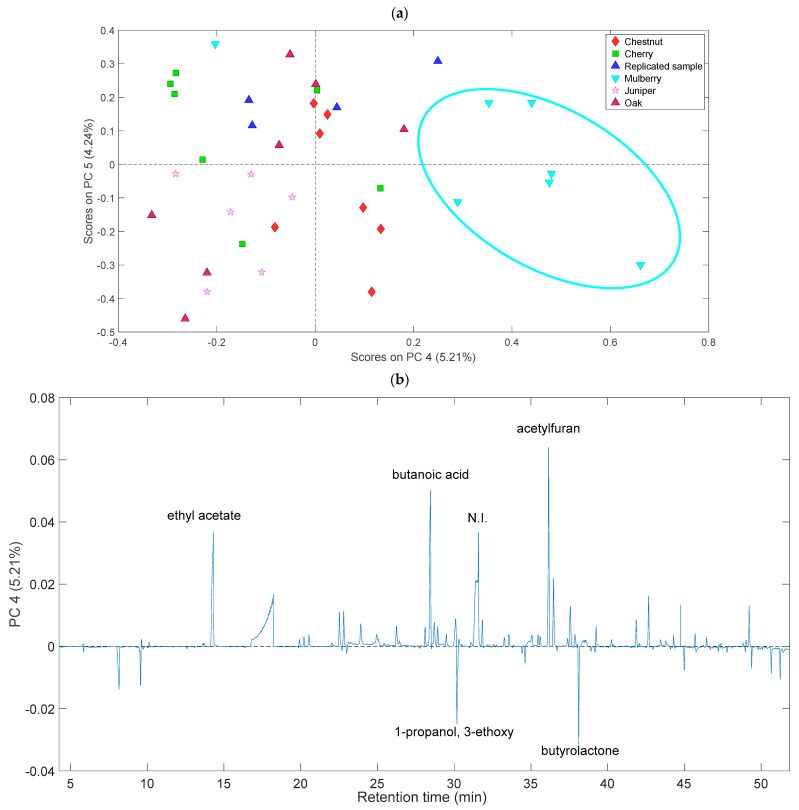
(**a**) PC4 vs. PC5 score plots of the vinegar samples. Scores are colored according to the battery of origin. Ellipse was used exclusively to improve the visualization of results. (**b**) PC4 loading plots.

In order to have a better overall overview of the variation between the samples according to barrels/wood, PC4 vs. PC5 score and loading plots (Figure 4a,b) were investigated as well.

The scores plot for PC4 (Figure 4a) pointed out a clear differentiation of almost all mulberry samples, with positive scores on this component. Examining the corresponding loadings plot (Figure 4b), it was possible to identify potential contributors to this differentiation. In particular, positive loadings on PC4 suggested that ethyl acetate, butanoic acid, and acetylfuran could be likely more abundant in the mulberry samples compared to others. Finally, since PC5 did not seem to show differentiation among the samples, the results associated with this component were not discussed.

## 3. Materials and Methods

### 3.1. Sampling

Five sets of batteries were sampled from two different producers A (Acetaia del Cristo, Modena, Italy) and B (Acetaia Lancellotti, Modena, Italy) strictly adhering to traditional production procedures. Each series comprised six barrels. The first barrel (1) contained the youngest ABTM sample, and the last barrel contained the oldest. Some batteries had a sample, ABTM, used for sale and commercializing (23). In this context, the term “vinegar sample” is broadly used to indicate samples belonging to any of the barrels of the battery. The samples and their corresponding barrel characteristics are listed in Table 1.

To assess the reproducibility of the measurements across the entire duration of the study, the same vinegar sample, belonging to barrel 3 of the mulberry battery, was repeated four times (one for each measurement session). This sample is referred to as “QC” in the results and discussion section. The other samples were replicated once.

### 3.2. Determination of Volatile Compounds by HS-SPME-GC/FID and HS-SPME-GC/MSq

A Solid-Phase Micro-Extraction (SPME) holder (Supelco, Inc., Bellefonte, PA, USA) was used to conduct the SPME (HS) analysis manually with an SPME composed of carboxen/polydimethylsiloxane (CAR/PDMS, 75 μm film thickness). The fiber was previously conditioned following the instructions of the supplier. The samples were prepared by inserting 1 mL of ABTM in a 10 mL flask. The prepared sample was then thermostated at 30 °C in an oven, exposing the SPME fiber to the headspace for a duration of 30 min. Subsequently, the fiber was thermally desorbed in the hot injection port of the gas chromatographic system. All samples were analyzed in an HP 6890 Agilent Technologies gas chromatograph equipped with a flame ionization detector (FID). The GC-FID results were used for the multivariate analysis.

The injector was heated at 270 °C and the analyses were conducted in splitless mode, to obtain maximum sensitivity of response. An HP-624 column (60 m × 0.25 mm i.d., 1.40 μm film thickness; J&W Scientific, Folsom, CA, USA) was used. Helium, with a flow rate of 1.2 mL/min, was employed as the carrier gas. After 3 min, the split valve was opened and the gas saver option was activated, setting a flow rate of 30 mL/min. The temperature ramp program is reported in Table 2.

For compound identification, only a selected subset of samples was further analyzed by gas chromatography-mass spectrometry (GC-MS) due to time and cost constraints. In particular, a 6890N Agilent Technologies gas chromatography coupled to an MSD 5973N quadrupole mass analyzer (Agilent Technologies, Santa Clara, CA, USA) was used with the same sample preparation procedure, type of fiber, column, and chromatographic conditions described above. The injector was heated at 270 °C and the transfer line was heated at 270 °C. The quadrupole and source temperatures were maintained at 150 and 230 °C, respectively. Electron ionization mass spectra in the full-scan mode were recorded at 70 eV electron energy. The range of mass-to-charge ratio (*m*/*z*) spanned by the spectrometer was 15–300 amu for the first 40 min of the GC analysis, and 35–450 amu until the end of the run. Chromatograms and mass spectra were analyzed using Enhanced ChemStation software (version E.02.02.1431, Agilent Technologies, Santa Clara, CA, USA). The identification of volatile compounds was achieved by matching the mass spectra with the data system libraries (NIST08 and WILEY7n). Some analytes of major interest were identified by comparing their mass spectra and retention time with those of their respective pure standards (ethanol, ethyl acetate, acetic acid, furfural, 5-methylfurfural), analyzed under the same operating conditions used for the samples.

### 3.3. Reagents and Standards

Ethanol, acetic acid, and ethyl acetate were obtained from Carlo Erba, Milan (Italy). Furfural and ethyl acetate were obtained from Sigma-Aldrich, distributed by Merck KGaA, (Darmstadt, Germany).

### 3.4. Multivariate Analysis

The obtained chromatographic signals were analyzed by means of Principal Component Analysis. This approach not only allows for the identification of similarities, differences, or trends among the investigated samples but also enables the identification of signal regions (loadings of the principal components) that are most relevant in describing the characteristics of the studied matrix, in a fully exploratory context.

As the variation in signal intensity within the chromatograms reflects roughly the presence of both major and minor constituents, it was essential to employ a data pretreatment procedure capable of making the different chromatographic regions comparable in influencing the developed statistical models.

In particular, two different blocks were defined (labeled B1 and B2 in Figure 1a), each including peaks of similar variation together with some baseline regions (in order to avoid up-weighting of noise).

The weights, w_i,B_, to be assigned to each variable in a given block, B, are defined as Equation (1) [26]:

(1) $$w_{i,B}=\sqrt{\frac{\mathrm{SSTOT}}{\mathrm{SSBLOCK}\cdot \mathrm{nBLOCK}}}$$

where SSTOT is the total sum of squares over all i variables, SSBLOCK is the sum of squares over the i variables belonging to the given block and nBLOCK is the number of variables inside the block. In this way, both blocks of data are given an equal chance of influencing data variance.

PCA was carried out by using PLS_Toolbox 8.9.2 software (Eigenvector Research Inc., Manson, WA, USA) for MATLAB^®^ (Matlab version: 9.13.0, R2022b, Natick, MA, USA: The MathWorks Inc.; 2022).

## 4. Conclusions

The present study focused on the evolution of the aroma profile of Aceto Balsamico Tradizionale di Modena PDO (ABTM) aged using barrels of different types of woods. The entire sequence of operations that characterizes the production of ABTM, combined with the quality of the raw materials used as well as the skill of the artisans, are undoubtedly important factors in determining the final chemical and physicochemical properties of the product. The overlapping of physical, chemical, and biochemical phenomena gives rise to a complex labyrinth of transformations, where each component is interconnected with others to create a unified framework of aromas and flavors. In addition, the type of wood used for barrels could affect the final aroma characteristics too. In order to investigate these influences, the aromatic fractions of several vinegars coming from single-wood barrels were chemically characterized using a solid-phase micro-extraction technique, SPME, coupled with gas chromatography-mass spectrometry, GC/MSq. Specifically, batteries made of cherry, chestnut, mulberry, juniper, and oak were examined. It was possible to monitor the evolution of the volatile fraction through a multivariate approach that allowed samples to be characterized according to different aging times and different battery origins. Although it was not possible to identify, through the employed method, molecules directly connected to the woody essence, some similarities emerged between samples from mulberry and cherry barrels and between those from barrels of juniper and oak. However, the highly complex and variable nature of the production process further limits the generalizability of these findings. Future studies should aim to analyze a larger and more diverse set of samples to validate this preliminary observation.

## Figures and Tables

**Table 1:** ABTM samples characteristics.

Barrel Number	Barrel Material	Producer
6	Mulberry	A
5	Mulberry	
4	Mulberry	
3	Mulberry	
2	Mulberry	
1	Mulberry	
ABTM	Mulberry	
6	Chestnut	A
5	Chestnut	
4	Chestnut	
3	Chestnut	
2	Chestnut	
1	Chestnut	
ABTM	Chestnut	
6	Juniper	A
5	Juniper	
4	Juniper	
3	Juniper	
2	Juniper	
1	Juniper	
ABTM	Juniper	
6	Cherry	A
5	Cherry	
4	Cherry	
3	Cherry	
2	Cherry	
1	Cherry	
ABTM	Cherry	
6	Oak	B
5	Oak	
4	Oak	
3	Oak	
2	Oak	
1	Oak	
ABTM	Oak	

**Table 2:** GC method employed.

Ramp Rate (°C/min)	Temperature (°C)	Hold Time (min)	Elapsed Time (min)
	30	1.00	1.00
3.00	150	0.00	40.00
8.00	260	14.50	28.25
			Tot: 69.25

## Data Availability

The data presented in this study are available in article and Supplementary Materials.

## Supplementary Materials

The following supporting information can be downloaded at: https://www.mdpi.com/article/10.3390/molecules29112647/s1, Table S1: HS-SPME-GC/MSq results.

## Abbreviations

ABM	Aceto Balsamico di Modena
ABTM	Aceto Balsamico Tradizionale di Modena
GC-FID	Gas Chromatography-Flame Ionization Detector
GC-MS	Gas Chromatography-Mass Spectrometry
HS-SPME	Headspace Solid-Phase Micro-Extraction
PCA	Principal Component Analysis
PDO	Protected Designation of Origin
PGI	Protected Geographical Indication
TSG	Traditional Specialties Guaranteed
