# Supplementary material for: Exploring the Impact of Various Wooden Barrels on the Aromatic Profile of Aceto Balsamico Tradizionale di Modena by Means of Principal Component Analysis
Source: Molecules. 2024 Jun 4;29(11):2647. doi: 10.3390/molecules29112647 (PMC11173617; doi:10.3390/molecules29112647)
Supplement: Supplementary file 1 [file molecules-29-02647-s001.zip › molecules-3011613-SI.pdf]

Table S1. HS-SPME-GC/MSq results

Retention Time (min)	Analyte	Sample 3	Sample 6
7.957	Ethanol	X	
9.548	Ethyl formate	X	
13.871	Vinyl acetate	X	X
14.384	Ethyl acetate	X	X
17.556	Acetic acid	X	X
20.537	Propyl acetate	X	
22.413	2-Propen-1-ol, 2-methyl-, acetate		X
22.833	Propane, 1-methoxy-2-methyl-	X	X
23.361	Isoamyl alcohol	X	
23.563	1-Butanol, 2-methyl-	X	
25.540	Ethyl butanoate	X	
26.557	Isobutyric acid	X	X
28.456	Butanoic acid	X	
28.812	2,3-Butanediol	X	X
30.229	1-Propanol, 3-ethoxy-	X	X
30.559	Furfural	X	X
30.937	Isopentyl acetate	X	
31.122	2-Methylbutyl acetate	X	
31.981	Isovaleric acid	X	X
32.303	Butyric acid, 2-methyl-	X	X
33.870	2,3-Butanediyl diacetate		X
35.065	2-Cyclopentene-1,4-dione	X	
35.277	Acetylfuran	X	X
38.192	Butyrolactone		X
38.299	Benzaldehyde	X	X
38.802	Furfural, 5-methyl-	X	X
39.890	Hexanoic acid	X	X
41.011	1,2-Propanediol, diacetate	X	X
42.315	Phenol	X	X
42.589	2,3-Butanediyl diacetate	X	X
42.709	Ethanone, 1-(2-methyl-1-cyclopenten-1-yl)-	X	X
43.098	Benzaldehyde	X	X
43.353	2,3-Butanediyl diacetate	X	X
43.661	Pentanoic acid, 4-oxo-, ethyl ester	X	X
43.834	2,5-Furandione, 3,4-dimethyl-		X
44.514	Nonanal	X	
44.955	1,3-Propanediol, diacetate	X	X
46.349	Phenylethyl Alcohol	X	X
47.095	Benzyl Acetate	X	X
47.811	Octanoic Acid	X	X
49.462	Ethyl benzoate	X	
49.850	Phenethyl propionate	X	X
51.245	5-Hydroxymethylfurfural		X
51.831	5-Acetoxymethyl-2-furaldehyde		X
